# Integrating Multiclass Light Weighted BiLSTM Model for Classifying Negative Emotions

**DOI:** 10.1155/2022/5075277

**Published:** 2022-07-30

**Authors:** Manisha Bhende, Anuradha Thakare, Bhasker Pant, Piyush Singhal, Swati Shinde, Betty Nokobi Dugbakie

**Affiliations:** ^1^Marathwada Mitra Mandal's Institute of Technology, Pune, India; ^2^Pimpri Chinchwad College of Engineering, Pune, India; ^3^Department of Computer Science & Engineering, Graphic Era Deemed to be University, Dehradun, Uttarakhand, India; ^4^Department of Mechanical Engineering, GLA University, Mathura, UP, India; ^5^Department of Computer Engineering, Pimpri Chinchwad College of Engineering, Pune, India; ^6^Department of Chemical Engineering, Kwame Nkrumah University of Science and Technology, KNUST, Ghana

## Abstract

With the continuous development of social networks, Weibo has become an essential platform for people to share their opinions and feelings in daily life. Analysis of users' emotional tendencies can be effectively applied to public opinion control, public opinion surveys, and product recommendations. However, the traditional deep learning algorithm often needs a large amount of data to be retained to obtain a better accuracy when faced with new work tasks. Given this situation, a multiclassification method of microblog negative sentiment based on MAML (model-agnostic metalearning) and BiLSTM (bidirectional extended short-term memory network) is proposed to represent the microblog text word vectorization and the combination of MAML and BiLSTM is constructed. The model of BiLSTM realizes the classification of negative emotions on Weibo and updates the parameters through machine gradient descent; the metalearner in MAML calculates the sum of the losses of multiple pieces of training, performs a second gradient descent, and updates the metalearner parameters. The updated metalearner can quickly iterate when faced with a new Weibo negative sentiment classification task. The experimental results show that compared with the prepopular model, on the Weibo negative sentiment dataset, the precision rate, recall rate, and F1 value are increased by 1.68 percentage points, 2.86 percentage points, and 2.27 percentage points, respectively.

## 1. Introduction

Artificial intelligence is a subclass of data science that aims to develop smart computers capable of doing a wide range of tasks, which would ordinarily normally require human comprehension. These intelligent devices can reflect on their prior experiences and information, evaluate their environment, and take necessary measures. Human intelligence is defined as the ability to learn from previous experiences, adapt to new situations, handle abstract thinking, and transform one's own surroundings using the knowledge gained. Human intelligence differs from artificial intelligence in that AI aims to create technologies that can mimic human behaviour and perform humane activities, whereas human intelligence aims to adapt to new situations by combining various cognitive states. Humans rely on their brains' memories, computing capability, and ability to think, but AI-powered computers rely on facts and directions input into the system. The ability to learn and comprehend from numerous situations and past experiences is the foundation of human intellect. However, because AI cannot think, it lags in this field. The goal of the study is to perform sentiment analysis using MAML and BiLSTM for negative sentiment investigation among a smaller number of samples when compared to other formats like deep learning and other ML techniques.

Weibo is a platform for sharing and commenting, and more and more individuals are using it to openly express their thoughts and feelings. Weibo has evolved into one of the most important platforms for people to communicate and share their feelings. The enormous amount of data generated serves as a foundation for text sentiment classification. Researchers are increasingly interested in analyzing the emotional patterns of Weibo users. At the same time, it has become a natural language processing research hotspot. Text sentiment analysis is a concept. It is a technique of examining, reasoning, and summarizing emotionally subjective texts to mine the emotional polarity of users in textual information, and it was first suggested in literature [1]. Sentiment analysis on user microblogs is useful for quickly determining the public's emotional state and obtaining public views and attitudes. It can be applied to a variety of fields, including product evaluation, movie evaluation, and news recommendation.

The majority of traditional sentiment classification techniques is rule based or machine learning based. Machine learning-based methods first create a training set by manually labelling a portion of the data and then extract and learn features from the training set data. Rule-based methods rely on experience or expert opinions to obtain statistical features, sentiment dictionaries, and other tools to classify text sentiment, to create a classification model and then apply it to data with unknown labels in order to do sentiment categorization. However, when the amount of data grows, the characteristics of text information become more complicated, exposing the inadequacies of these two methodologies. They rely on complex feature engineering far too much. Deep learning's emergence has opened up new possibilities for natural language processing tasks. For example, literature [2] introduced the RNTN (recursive neural tensor network) model for binary sentiment categorization based on recurrent neural networks; literature [3] analyzed the sentiment polarity of text using a long short-term memory network; literature [4] suggested aspect-level sentiment categorization, literature [5] employed the MAN (mutual attention neural network) model, used GCN (graph convolutional networks) to model sentiment dependencies, and subsequently did sentiment categorization. A study presents a method for detecting sarcasm that uses the PCA algorithm, the K-means algorithm, and ensemble classification [6]. Deep learning models can train excellent features for target problems automatically, and they have done well in binary text sentiment classification tasks. In multiclassification jobs, however, there is still space for improvement.

Unlike the majority of the existing literature focusing mainly on ML and deep learning for sentiment analysis, this study talks about the application of model-agnostic metalearning (MAML) bidirectional long short-term memory (BiLSTM) for analyzing the sentiments posted by people on Weibo. Given the variety of sentiment classification challenges and the fact that deep learning models sometimes need a significant amount of data retraining to produce visible results when faced with new classification problems, this study will swiftly adapt to metalearning. In the event of limited samples, deep learning of new tasks is required. In addition to the techniques, a multiclassification method based on MAML and BiLSTM for Weibo negative sentiment is presented. A Weibo negative sentiment dataset with 13 categories is created using current sentiment analysis resources and data. Text input with negative sentiment is encoded, a semantic representation is built, and parameters are taught. This study successfully enhances the sentiment analysis performance of the system for microblog text in small samples by integrating BiLSTM and MAML. The following are the study's key contributions: (1) a new Weibo microblog is created utilizing the current Weibo corpus and crawled Weibo data. Experiments are conducted using the Weibo negative sentiment dataset. (2) Based on MAML and BiLSTM, a multiclassification technique for Weibo negative sentiment is developed. The MAML metalearning framework is added to the classic BiLSTM in this model. Designing similar tests validates the new model's performance in the microblog sentiment multiclassification job. (3) It has been shown via the design of related studies that the use of MAML in the microblog sentiment classification task may increase the task's performance. The performance of our model outperforms that of general deep learning models, according to experimental findings.

## 2. Related Work

### 2.1. Metalearning

A fundamental quality that separates human intelligence from artificial intelligence is the capacity to learn fast. Although classical deep learning has had a lot of success in data mining, classifiers trained this way generally perform badly when just a limited quantity of data is provided or when demands change. When learning new tasks, this is mostly due to a lack of effective past knowledge. Literature [7] established the notion of metalearning, which overcomes the aforementioned concerns by accumulating metaknowledge from the learning experience of multiple tasks [8–10]. The inductive bias provided by metalearning allows the model to generalize across various tasks, resulting in rapid learning algorithms that are efficient for sampling [10]. Many deep learning studies based on metalearning have been published in the past. Literature [11], for example, looked at the usage of recurrent neural networks (RNNs) to perform algorithmic problems. They tried using an LSTM-based metalearner; however, the results revealed that the LSTM design is not ideal for this sort of work. They next created a more complicated RNN architecture, in which the LSTM controller was linked to an external repository that could read and write, and proved that MANNs outperformed LSTMs. The authors [12] compared LSTM and MANN metalearners for few-shot image categorization, confirming the LSTM architecture's shortcomings. MANNs, on the other hand, have a complicated memory addressing design that is difficult to train. In a few-shot classification context, where the usual learner is a neural network-based classifier, authors [13] utilized a similar LSTM metalearner. The standard learner starting parameters are learned to adapt to fast gradient changes, and the LSTM metalearner is trained as an optimization method customized to the metalearning job. Authors [14] investigated a particular example, in which the metalearner must update the classifier using gradient descent, and showed that a reduced model (dubbed MAML) may obtain equal results. Authors [15] investigated a more complex weight updating approach that yielded slight improvements in few-shot classification performance. Authors [16] introduced a unique metalearning approach for encoder-decoder mode in their most recent study. They devised a method for learning iterative updates to encode contextual information into task representation, as well as a metalearner. During the updating phase, the model is improved, and the final representation is utilized to limit the decoder to make predictions on unlabeled data, resulting in the best performance on the benchmark dataset tiered ImageNet [17]. On the basis of MAML, the authors [15] established the idea of “task robustness.” They reasoned that metalearning approaches were only effective in a few particular distribution tasks, thus they recreated MAML using a new formula, resulting in a stable model.

### 2.2. Weibo Sentiment Classification

The metalearning layer's goal is to let the model learn new tasks rapidly with only a modest amount of additional data. Metalearning allows one to direct new learning activities based on the existing experience, permitting one to memorize. MAML is a good method that can easily be integrated with neural networks and applied to a variety of different loss functions. With its distinctive traits, Weibo has drawn numerous experts to explore text sentiment analysis since its inception. The majority of research is still focused on rule-based approaches, machine learning-based methods, and deep learning-based methods. Authors [18], for example, used the similarity calculation method to expand the knowledge base of emotional words, built a bilingual inspirational dictionary, and used the Gauss mixture model and k-nearest neighbor algorithm of symmetric relative entropy to realize sentiment classification of microblog texts. Authors [19] investigated the characteristics of microblog text information and combined machine learning algorithm and feature selection to classify microblog texts. However, because traditional approaches depend too much on feature engineering and need a large workforce, an increasing number of academics are turning to deep learning methods to handle emotional difficulties. The authors [20] proposed building a CharSCNN (character to sentence convolutional neural network) model based on words, using the letters that makeup words as units. The CharSCNN model is based on CNN and employs two parallel convolutional layers to learn the structural aspects of words and the semantic features of sentences, which completely captures CNN's capacity to abstract and extract local text characteristics. In short text sentiment binary classification, the model yields promising results. The authors [21] presented an attention-based BiLSTM neural network to qualitatively collect useful semantic information. Sentiment analysis on short texts is performed by learning semantic properties from two directions of phrases. Experiments on the Stanford tree graph dataset and the movie review dataset show that the model increases LSTM performance by 3% over the nonattention model. The authors [22] built a novel feature set including statistical knowledge, linguistic knowledge, emotional knowledge, emotion conversion rules, and word embedding as feature vectors and showed that using this feature set may significantly increase the classification accuracy of neural network models. Kaur Shabaz (2019) attempted to retrieve information from social media sites using the association mining technique to learn about people's opinions on current and upcoming topics. They employed the KKSSA technique in their study to do sentimental analysis on real-time data gathered from social media [23]. The authors [24] suggested a novel model Trans-CAP (transmission capsule network) for sentiment categorization at the aspect level. Capsule networks are commonly utilized in classification and feature extraction applications. Capsule networks can preserve more information than CNN. They create a dynamic routing approach in this model that captures sentence-level semantic representations as semantic capsules from aspect-level and document-level data and allow it to adapt to a transfer learning framework.

## 3. Model Framework Based on MAML and BiLSTM

### 3.1. Construction of the Weibo Negative Sentiment Dataset

A new dataset is constructed to meet the experimental needs: Weibo negative sentiment dataset. This study integrates the microblog text data obtained by the crawler program with the existing microblog data resources on the Internet and receives a total of 120566 pieces of original data. Then, the Sina Weibo text data were annotated by manual annotation. The annotation team consisted of 8 people, all laboratory members, with relevant professional knowledge and average cognitive ability. To ensure the availability of the data, two rounds of cross-labelling are adopted. Each person is responsible for labelling a type of label data. After the labelling is completed, the second member will check it. When there is any objection to the labelling result, the third member will confirm the final construction. A multilabel dataset of 19,500 pieces of data was created.

### 3.2. Specific Algorithm and Model Architecture

**Figure 1:**
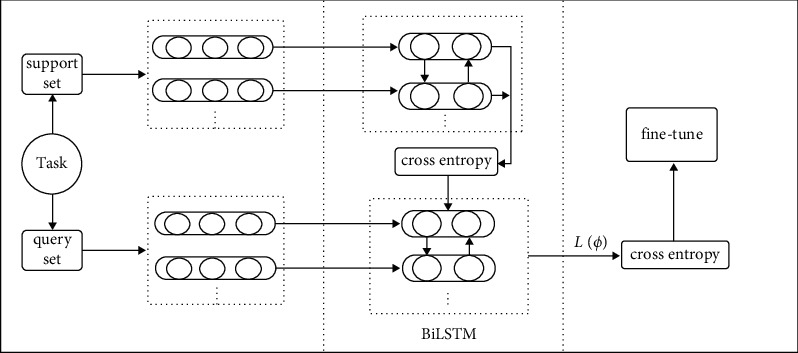
Model architecture.

The interdependencies between words must be considered while analyzing microblog text data. As a result, this work uses the BiLSTM algorithm, which can extract features from the text while simultaneously obtaining semantic information from the environment. In the case of long-term reliance information, LSTM has a significant learning ability. However, the typical LSTM model could only identify positive semantic relationships in the content and not reverse semantic features. Thus, the BiLSTM model is used in this study. This study introduces a metalearning method based on BiLSTM bidirectional extended short-term memory network, and a multiclassification process of Weibo negative emotions based on MAML and BiLSTM is constructed. The overall architecture is shown in Figure 1.

#### 3.2.1. Word Vector Representation Layer

The word vector representation layer serves as the model's input. Before vectoring the data needed for the experiment, the samples are picked at random based on the experimental conditions in few-shot learning. For example, the 5-way 5-shot must collect data from 5 categories, and the number of items in each variation must be more than 5. These samples refer to it as a task and divide it into two parts: a support set and a query set. The support set has 5 types of models, with 5 pieces for each type, whereas the query set contains all of the remaining 5 types of models, with a number that generally surpasses 5 to increase the model's generalization capacity. The data vectored representation step comes next. This phase necessitates the use of a dictionary ddn. The dictionary is produced from a big corpus after word vectorization training and may be used immediately, where *d* is the vector word's dimension and *N* is the number of words. In this study, the word globalization representation is to preprocess each data into a text sequence *T* = *w*_1_, *w*_*w*_,…, *w*_*n*_ and then match each word *w* with a vector word in the dictionary, to eventually generate the full word vector representing a series of text.

#### 3.2.2. BiLSTM Layer

For microblog text data, the dependencies between words need to be considered. Therefore, this study adopts the BiLSTM algorithm, which can obtain the semantic information of the context simultaneously and more fully extract the features contained in the text.

LSTM happens to have a strong learning ability for long-term dependency information. Still, the general LSTM model can only capture the positive semantic relationship in the text and cannot obtain reverse semantic information. Therefore, this study adopts the BiLSTM model. BiLSTM consists of two LSTM models, positive and negative. It is assumed that the input text sequence is *X* = {*x*_1_, *x*_2_,…, *x*_*n*_}, where *x*_*i*_(*i* = *i*_1_, *i*_2_,…, *i*_*n*_) is the word vector. The forward LSTM can learn the semantic relationship from *x*_1_ to *x*_*n*_, and the same is true for the reverse LSTM so that context information can be obtained simultaneously. LSTM consists of cell units and has three gate structures to control the network's data: forgotten gate, input gate, and output gate. In the specific process, the first pass is the forget gate, which is used to retain the previous hidden state selectively. The formula is as follows:

(1) $$\begin{array}{c} g_{x=\sigma \left(Z_{f}.\left[rt-1,xt\right]+bf\right)}. \end{array}$$

Among them, *Wo* is the output gate weight, and *ct* is the cell unit state at the current moment. In the bidirectional long-term memory network, the input *n*-dimensional word vector passes through the two cell units before and after. The output information at each moment is represented by the output connection of the two cell units, which is expressed as $\tilde{o}t$ = [*ot*, *o*′*t*], and at the same time, the semantic information of the context is considered:

(2) $$\begin{array}{c} it=\sigma \left(Wi\cdot \left[ht-1,xt\right]+bi\right),\\ C^{∼}t=\tanh\left(W_{c}.\left[rt-1,xt\right]+mn\right). \end{array}$$

In this model, the data of the support set in each task are trained by the BiLSTM layer, and the learner parameters are continuously learned and updated:

(3) $$\begin{array}{c} ot=\sigma \left(Wo\cdot \left[ht-1,xt\right]+bo\right),\\ ht=ot*\tanh\left(rt\right). \end{array}$$

After the update, the data of the query set are introduced by this layer, and the loss is calculated. Through the above training process, the required information can be provided for the learning process of the metalearner.

#### 3.2.3. Metatraining Layer

The purpose of the metalearning layer is to enable the model to quickly learn new tasks from a small amount of further data. Through metalearning, one can use previous experience to guide new learning tasks, allowing one to remember. MAML is a good algorithm that can be easily combined with neural networks and used for various other loss functions.

MAML directly optimizes the initialization parameters of the learner so that when faced with a new task, the learner can achieve the maximum generalization performance on the new job after updating the parameters with only one or more gradient descent steps calculated with a small amount of data. The specific process is to extract data from the data set and divide it into multiple tasks called *Ti*. After a gradient descent on the support set of T1, the parameter *θ* of the current most suitable job is obtained and then tested on the query set of T1 to accept the loss LT1 *f* (*θ*) of the current task. Next, after extracting the second task, the loss of the corresponding job occurs until the defeat of duty *Tn* is obtained, and the sum of the failures of these tasks is taken:

(4) $$\begin{array}{c} L\left(\emptyset \right)=\sum_{n=1}^{N}LTnf\left(\theta -n\right), \end{array}$$

where *f* (*θ*) is the loss function and the cross-entropy loss function is used in this study. Finally, the sum of the test errors obtained on different tasks is used as the optimization objective of the metalearner, and the parameters are updated by the gradient descent method. Finally, a set of initialization parameters are applied to fine tune to get the model expected in this study. As a result, it can achieve good performance with only a small amount of data after one or more gradient descents on Weibo negative sentiment classification.

## 4. Experiments and Analysis

### 4.1. Dataset

The experimental data used in this study are a new self-labeled dataset: Weibo negative sentiment dataset. The dataset is divided into two parts: a training set and a test set, and each piece of data includes sentiment labels and text content. There are 13 kinds of emotional tags in total, and each type of tag has 1500 pieces of data, totaling 19500 pieces. Various sentiment labels and data samples are listed in Table 1. This study conducts comparative experiments in two dimensions of different models and data sets with varying division ratios.

First, according to the experimental settings of previous metalearning research, in the model comparison experiment, the set training set includes eight types of data, with a total of 12,000 items. The test set consists of 5 categories, with 7500 items. On this basis, comparative experiments of different models are carried out.

In addition, because the dataset contains few categories, based on the method in this study, different types of proportions are randomly selected as the training set and the test set for comparison experiments, and the influence of varying category proportions in the current data set on the experimental results is studied.

When constructing the microblog text data into word vectors, this study uses the IMDB data set as the pretrained word vector dictionary, the training process uses the GloVe model, and the parameters are all default settings. When segmenting the microblog data, the python Chinese word segmentation component jieba is used for word segmentation. In addition, this study also conducts a series of microblog text preprocessing, such as traditional and simple conversion, removal of stop words, and elimination of meaningless symbols. Finally, through the word embedding process, a word vector space containing 46357 words is obtained, and the word vector dimension of each word is 300.

### 4.2. Experimental Parameter Settings

The same parameters are used in the two experiments in this study, and the specific experimental parameter settings are listed in Table 2.

### 4.3. Experimental Comparison Model

*MNB Model (Multinomial Naive Bayes)* [25]. It is a representative of traditional machine learning and has achieved excellent results in a large number of multiclassification tasks.

*MCNN Model (MultiChannel Convolutional Neural Network)* [26]. In the existing research that uses deep learning to identify texts, the CNN uses so-called convolutional filters, which automatically identify characteristics that are appropriate for the task at hand. Convolutional filters, for example, may capture fundamental syntactic and semantic aspects of sentimental phrases if we utilize the CNN for sentiment classification. It has been demonstrated that a single convolutional layer, consisting of a collection of convolutional filters, may obtain similar results without any parameter adjustment [27]. The MCNN model uses a multichannel convolutional neural network to learn the representation of text word vectors, thereby realizing the classification of text sentiment. It is an early application of neural networks to the sentiment analysis task model.

*BiLSTM Model*. This model adopts the LSTM network structure described in literature [28], based on the word vector representation of text, combined with a bidirectional long-short-term memory network to capture contextual information for classification.

*Matching Nets Model* [29]. This model combines the popular attention structure and memory network to build a fast-learning network, including the feature extraction network, memory network, distance metric network, and classification network using the attention module. In the training phase, we directly imitate the situation where there are only a few labeled samples in the testing phase. During the model training process, a single support set and a test sample will be used as inputs to perform features at the same time.

*Metalearn LSTM Model* [13]. The author of this model found that the update rules of LSTM are similar to the update rules of the general gradient descent algorithm, so a metalearner LSTM was trained, and the unit state update formula of LSTM was used to learn the parameter update rules, thereby training the neural network.

### 4.4. Analysis of Experimental Results

#### 4.4.1. Model Comparison Experimental Analysis

**Figure 2:**
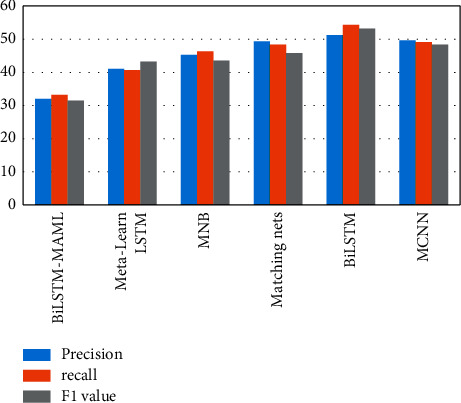
5-way 1-shot experimental results.

**Figure 3:**
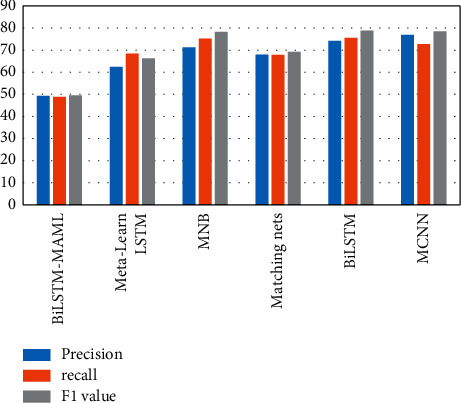
5-way 5-shot experimental results.

The experimental results of this algorithm and other algorithms on the same data set are shown in Figures 2 and 3 and Tables 3 and 4.

From the results in Figures 2 and 3, the neural network model is generally more suitable for Weibo negative sentiment classification than traditional machine learning. By comparing the experimental results of the MCNN and BiLSTM models, it is shown that the performance of the two models is similar when the number of samples is too small, and BiLSTM can simultaneously obtain contextual information of text data through backpropagation, which is more suitable for this task than MCNN. Matching nets is a standard model in few-shot learning and has good performance in sentiment multiclassification tasks. Compared with BiLSTM in the 5-way 5-shot experiment, the accuracy, recall, and *F*1 value are increased by 3.19 percentage points, 3.87 percentage points, and 4.68 percentage points, indicating that in the text sentiment multiclassification task, the memory enhancement network contains important information that can be preserved, but at the same time, a good learning strategy can keep performance improving.

The accuracy rate, recall rate, and *F*1 value of metalearn LSTM in the two experiments ranked second on the Weibo negative sentiment dataset. Overall, the performance of metalearn LSTM significantly exceeded the previous four models, indicating that the metalearning idea is more effective for Weibo negative sentiment classification task. Comparing the experimental results of BiLSTM-MAML and metalearn LSTM models shows that the BiLSTM-MAML model has the best performance on this dataset and has a noticeable improvement in the 5-way 5-shot experiment terms of accuracy and recall and *F*1 values increased by 1.68 percentage points, 2.86 percentage points, and 2.27 percentage points, respectively. It can be seen that when the dataset samples are emotional text data, the BiLSTM model can be used by the learner to promote the learning process of the metalearner more effectively, and it also fully proves the effectiveness of the method in this study.

#### 4.4.2. Experimental Analysis of Data Set Scale Division

**Figure 4:**
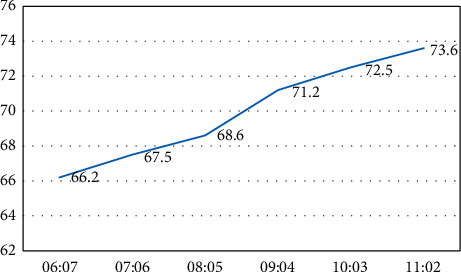
Data set division experiment.

The proportion of different categories in the dataset and the experimental results are shown in Figure 4.

After experiments, it was found that the classification accuracy dropped significantly when the number of sample categories in the training set was too small. For example, when the ratio of the training set to test set data was 6 : 7, the accuracy was only 60.48%, significantly lower than other ratios. The ratio was 8 : 8.5 and above, with the increase of the training set sample categories, the accuracy rate slightly increases, and the maximum gain is 0.66 percentage points. From the overall experimental results, in the case of sufficient training samples, the classification accuracy under multiple ratios approaches, the number of data set categories has little impact on the experiment, and the model has specific stability, as listed in Table 5.

## 5. Conclusion

This study proposes a multiclassification method of Weibo negative sentiment based on MAML and BiLSTM. Using existing sentiment analysis resources and data, a Weibo negative sentiment dataset containing 13 categories is constructed. Negative sentiment text data is encoded, semantic representation is constructed, and parameters are trained. The BiLSTM model implements the classification of negative emotions on Weibo and updates the parameters using machine gradient descent; the MAML metalearner calculates the sum of the losses from various training and courses, performs a second gradient descent, and updates the metalearner parameters. When faced with a new Weibo negative sentiment classification challenge, the upgraded metalearner can adapt quickly. On the Weibo negative sentiment dataset, the precision rate, recall rate, and *F*1 value all rose by 1.68%, 2.86%, and 2.27%, respectively, when compared to the prepopular model. By combining BiLSTM with MAML, this study effectively improves the sentiment analysis performance of the model for microblog text in the case of small sample learning. On the Weibo negative sentiment dataset, the model proposed in this study shows superior performance and certain stability and surpasses the current better models in multiple indicators, which proves the effectiveness of this study in Weibo negative sentiment analysis.

## 6. Future Scope

As deep learning advances further towards unsupervised learning, the utility of metalearning is gradually growing. Given the rapid advancement of deep learning, such a research study could have a wide range of applications. From basic more extensive hyperparameter search and elimination studies to computational adjustments or extensions, or simply exploration of even more futuristic methods to new datasets and contexts, the possibilities are endless.

## Figures and Tables

**Table 1:** Sample of microblog negative sentiment data set.

Emotional labels	Data sample
Sad	It's not easy to go home for New Year... tears
Ridicule	Sir, can you speak Japanese? despise
Nausea	This Li Bai is really disgusting…
Anger	The golden body that has not been delayed for six years was broken by a certain sister ∼ Now I am angry!
Envy	Annie's new avatar is a bug, I admit that I'm sour
Fear	I've been dreaming of being slashed across my face with a knife, it's so scary.
Personal attacks	This green tea bitch in Fenghui makes me sick to death
Related to yellow	Give me the Indian and American color map!
Disappointed	Hey, it's another night of work without sleep, too many things going on recently.
Concern	There is no Gang mutton knife-cut noodles … irritable.
Marketing information	Go home to grab tickets http//irctc
Pain	Come and help me, it hurts to be sick
Harmful information	Chen underground organization colluded with ukraine separatist forces

**Table 2:** Model parameter table.

Parameter	Illustrate	Value
Optimizer	Model optimizer	Adam
Train_Lr	BiLSTM learning rate during training	0.01
Meta_Lr	Metalearner learning rate	0.001
Batch_Size	Batch data	25
Dropout	Neuron dropout ratio	0.3

**Table 3:** 5-way 1-shot experimental results.

Model	Precision	Recall	*F*1 value
BiLSTM-MAML	32.02	33.2	31.5
Metalearn LSTM	41.05	40.65	43.25
MNB	45.25	46.3	43.5
Matching nets	49.36	48.36	45.8
BiLSTM	51.2	54.3	53.2
MCNN	49.6	49.1	48.36

**Table 4:** 5-way 5-shot experimental results.

Model	Precision	Recall	*F*1 value
BiLSTM-MAML	49.3	48.9	49.6
Metalearn LSTM	62.5	68.5	66.3
MNB	71.3	75.2	78.3
Matching nets	68	67.9	69.3
BiLSTM	74.3	75.6	78.9
MCNN	76.9	72.8	78.6

**Table 5:** Accuracy of different ratios of training and test data.

Training ' test	Accuracy
6 : 07	66.2
7 : 06	67.5
8 : 05	68.6
9 : 04	71.2
10 : 03	72.5
11 : 02	73.6

## Data Availability

The data shall be made available on request to the corresponding author.
